# Supplementary figures and images for: Ciliary length regulation by intraflagellar transport in zebrafish
Source: eLife. 2024 Dec 13;13:RP93168. doi: 10.7554/eLife.93168 (PMC11643619; doi:10.7554/eLife.93168)

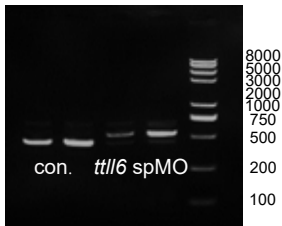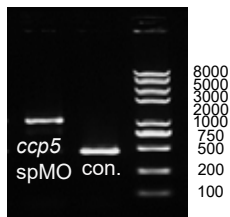

Supplement: Figure 4—figure supplement 3—source data 1. [file elife-93168-fig4-figsupp3-data1.zip › Figure 4-figure supplement 3-source data 1/Figure 4-figure supplement 3-source data 1.pdf]

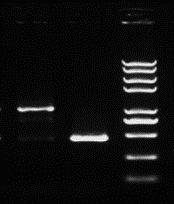

Supplement: Figure 4—figure supplement 3—source data 2. [file elife-93168-fig4-figsupp3-data2.zip › Figure 4-figure supplement 3-source data 2/CCP5 spMO.png]

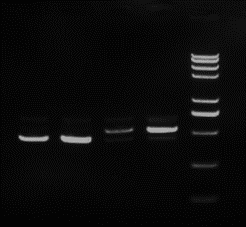

Supplement: Figure 4—figure supplement 3—source data 2. [file elife-93168-fig4-figsupp3-data2.zip › Figure 4-figure supplement 3-source data 2/ttll6 spMO.png]
