# Supplementary material for: Ciliary length regulation by intraflagellar transport in zebrafish
Source: eLife. 2024 Dec 13;13:RP93168. doi: 10.7554/eLife.93168 (PMC11643619; doi:10.7554/eLife.93168)
Supplement: Supplementary file 1. [file elife-93168-supp1.docx]

**Supplementary File 1**


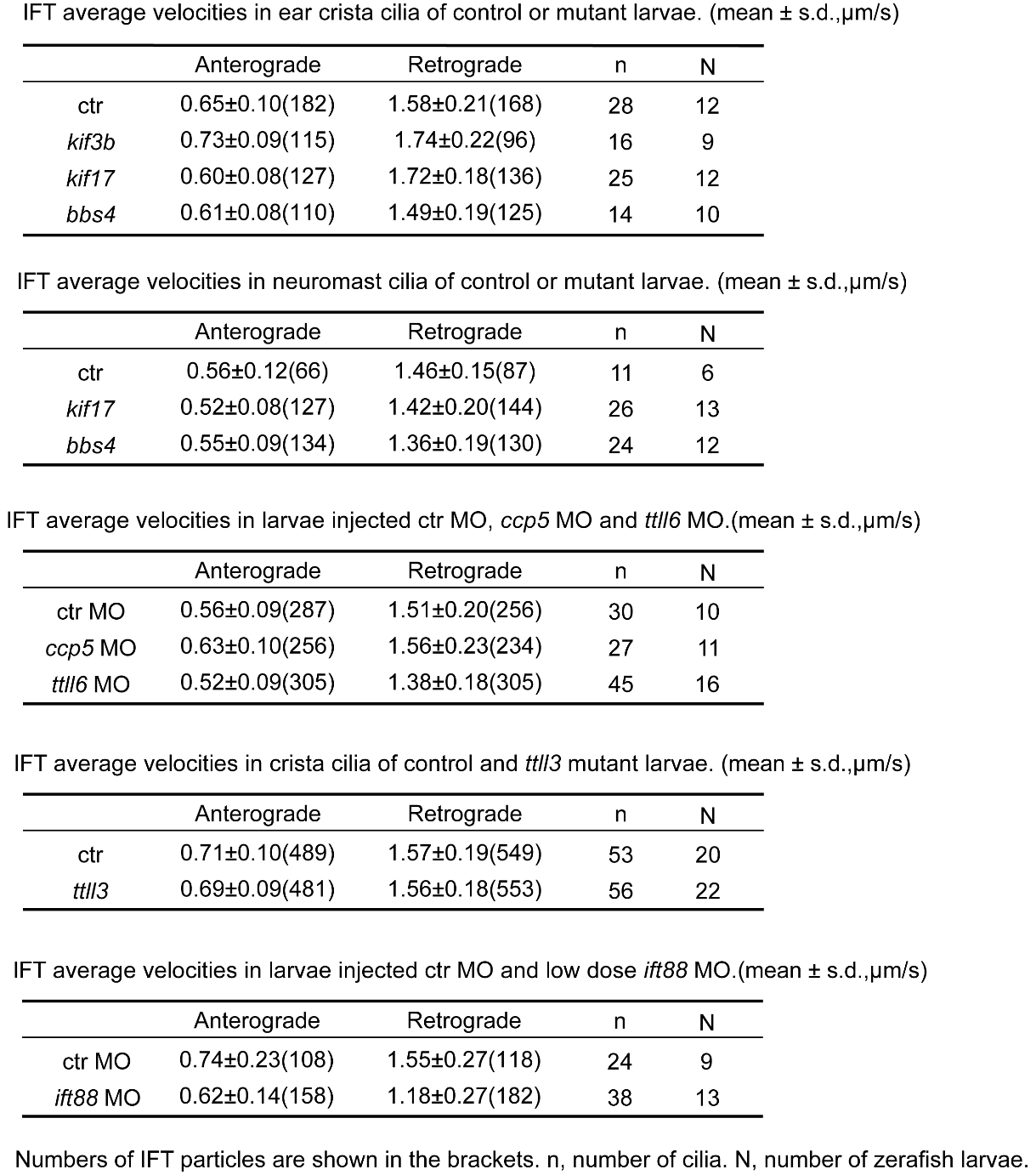
**Figure S1 Summary of average IFT velocities in different zebrafish muants or morphants**
