# Supplementary material for: Ciliary length regulation by intraflagellar transport in zebrafish
Source: eLife. 2024 Dec 13;13:RP93168. doi: 10.7554/eLife.93168 (PMC11643619; doi:10.7554/eLife.93168)
Supplement: Supplementary file 2. [file elife-93168-supp2.docx]

**Table S1 Detailed statistics**

| **Figure** | **Comparison** | | **Statistical test** | **P value** |
| --- | --- | --- | --- | --- |
| **Fig 3F** | Crista vs Neuromast | Antero | two-tailed unpaired Mann-Whitney test | P＜0.0001 |
|  |  | Retro | two-tailed unpaired Mann-Whitney test | P=0.6620 |
|  | Crista vs Pronephric duct | Antero | two-tailed unpaired Mann-Whitney test | P＜0.0001 |
|  |  | Retro | two-tailed unpaired Mann-Whitney test | P＜0.0001 |
|  | Crista vs Spinal cord | Antero | two-tailed unpaired Mann-Whitney test | P＜0.0001 |
|  |  | Retro | two-tailed unpaired Mann-Whitney test | P＜0.0001 |
|  | Crista vs Epidermal cell | Antero | two-tailed unpaired Mann-Whitney test | P＜0.0001 |
|  |  | Retro | two-tailed unpaired Mann-Whitney test | P＜0.0001 |
|  | Neuromast vs Pronephric duct | Antero | two-tailed unpaired Student’s t-test | P＜0.0001 |
|  |  | Retro | two-tailed unpaired Student’s t-test | P＜0.0001 |
|  | Neuromast vs Spinal cord | Antero | two-tailed unpaired Student’s t-test with Welch's correction | P＜0.0001 |
|  |  | Retro | two-tailed unpaired Mann-Whitney test | P＜0.0001 |
|  | Neuromast vs Epidermal cell | Antero | two-tailed unpaired Student’s t-test | P＜0.0001 |
|  |  | Retro | two-tailed unpaired Student’s t-test with Welch's correction | P＜0.0001 |
|  | Pronephric duct vs Spinal cord | Antero | two-tailed unpaired Student’s t-test | P＜0.0001 |
|  |  | Retro | two-tailed unpaired Mann-Whitney test | P＜0.0001 |
|  | Pronephric duct vs Epidermal cell | Antero | two-tailed unpaired Student’s t-test with Welch's correction | P=0.0389 |
|  |  | Retro | two-tailed unpaired Student’s t-test with Welch's correction | P＜0.0001 |
|  | Spinal cord vs Epidermal cell | Antero | two-tailed unpaired Student’s t-test with Welch's correction | P＜0.0001 |
|  |  | Retro | two-tailed unpaired Mann-Whitney test | p=0.2432 |
| **Fig 4B** | ctr vs *kif3b* mutant | Antero | two-tailed unpaired Student’s t-test | P＜0.0001 |
|  |  | Retro | two-tailed unpaired Mann-Whitney test | P＜0.0001 |
|  | ctr vs *kif17* mutant | Antero | two-tailed unpaired Student’s t-test with Welch's correction | P＜0.0001 |
|  |  | Retro | two-tailed unpaired Mann-Whitney test | P＜0.0001 |
|  | ctr vs *bbs4* mutant | Antero | two-tailed unpaired Student’s t-test with Welch's correction | p=0.0002 |
|  |  | Retro | two-tailed unpaired Mann-Whitney test | P＜0.0001 |
| **Fig 4F** | ctr vs *ttll3* mutant | Antero | two-tailed unpaired Student’s t-test | P=0.0021 |
|  |  | Retro | two-tailed unpaired Mann-Whitney test | ns |
| **Fig 4G** | ctr MO vs *ccp5* MO | Antero | two-tailed unpaired Student’s t-test | P＜0.0001 |
|  |  | Retro | two-tailed unpaired Student’s t-test | P=0.0239 |
|  | ctr MO vs *ttll6* MO | Antero | two-tailed unpaired Mann-Whitney test | P＜0.0001 |
|  |  | Retro | two-tailed unpaired Student’s t-test | P＜0.0001 |
| **Fig 5K** | ctr vs *ift88* MO | Antero | two-tailed unpaired Mann-Whitney test | P＜0.0001 |
|  |  | Retro | two-tailed unpaired Student’s t-test | P＜0.0001 |
